# A descriptive investigation of the impact of student research projects arising from elective research courses

**DOI:** 10.1186/s13104-016-1865-1

**Published:** 2016-01-27

**Authors:** Sam Harirforoosh, David W. Stewart

**Affiliations:** Department of Pharmaceutical Sciences, Bill Gatton College of Pharmacy, East Tennessee State University, Box 70594, Johnson City, TN 37614-1708 USA; Department of Pharmacy Practice, Bill Gatton College of Pharmacy, East Tennessee State University, Johnson City, TN USA

**Keywords:** Pharmacy student, Research, Elective, Pharmacy, Student pharmacist

## Abstract

**Background:**

Pharmacy academicians have noted the need to develop research skills in student pharmacists. At the Gatton College of Pharmacy, significant focus has been placed on the development of research skills through offering elective research courses. In order to evaluate the impact of participation in the research elective(s), we analyzed college records and surveyed faculty members with regard to the number of poster/podium presentations, published peer-reviewed manuscripts, and funded projects.

**Results:**

Student enrollment in the research elective sequence has increased over time and has resulted in 81 poster presentations, 14 podium presentations, and 15 peer-reviewed publications.

**Conclusions:**

Implementation of a research elective sequence and fostering of a research culture amongst the faculty and students has resulted in increased student engagement in research and related scholarly activities.

## Background

While student pharmacists may not be specifically required to conduct research as part of their professional program, the Accreditation Council for Pharmacy Education (ACPE) standards [1, 2] do highlight the necessity of molding students into self-directed critical thinkers. The Center for the Advancement of Pharmacy Education (CAPE) Education Outcomes 2013 [3] also highlight the need for this skill. Student engagement in research/scholarship increases critical thinking and additionally encourages pursuance of a research-related career [4]. The improvement of learning skills has also been stated as a benefit of engaging students in research projects [5, 6] and both faculty preceptors and students feel the process is a valuable experience for students [7–9].

Over the past decade, several publications have discussed research opportunities for pharmacy students and posited a need for increased emphasis on research and scholarship amongst United States pharmacy faculty members and increased development of pharmacist-researchers [6, 10–14]. Whereas many publications related to research/scholarship advancement have been penned by scholars at schools and colleges that are considered research-intensive (i.e., Carnegie classification of Very High Research Activity) [11], ACPE calls for all schools/colleges to “broaden the professional horizons of students in areas such as scientific inquiry, scholarly concern for the profession, and the relevance and value of research” [1]. Kehrer and Svensson recently stated, “Pharmacy researchers have the potential to contribute greatly to improving pedagogy as well as health care” [15].

Johnson et al. noted an increase in enthusiasm about post-graduate training among students who completed a summer research program [12]. Additionally, pharmacist graduates who pursue post-graduate training are typically required to conduct research as part of their American Society of Health-system Pharmacists (ASHP) accredited program as the accreditation standards require the institution be engaged in the continuous quality improvement process [16]. A survey of training sites for pharmacy residents revealed that directors of pharmacy strongly felt that resident involvement resulted in more research or scholarship completed. However, the pharmacist preceptors themselves felt slightly less strong about this statement, and many respondents agreed that residents need more guidance in the research process [17].

It is commonly recognized that this residency based research process may very well be the first attempt the new graduate has taken at conducting research and the level of preceptor ability in mentoring the resident in the research process may vary greatly from institution to institution [18]. Survey research has shown that student pharmacists see value in a required research project as part of their Doctor of Pharmacy curriculum and those students preparing to enter residency training are more likely than their colleagues to hold this view [9].

The need for graduates with basic research skills should be expected to increase as college of pharmacy deans anticipated growth in residency training over the 5 year period from 2010 to 2015, with 69.2 % of deans planning to add residents and/or fellows to their programs when asked in a 2010 survey [19]. This is supported by data from ASHP showing an increase from 2543 PGY1 and PGY2 programs in 2011 to 3690 in 2015 [20].

The potential to train students for these programs by involvement in the research process undoubtedly exists in most schools and colleges of pharmacy. However, the extent of involvement of students in research experiences is varied among pharmacy schools, and recent data are lacking. Data collected by Murphy et al. indicated that 25 % of surveyed schools required some form of research be completed by students and 57 % offered elective research opportunities to students, while 18 % of the schools did not provide any research-related program [11]. Whereas a majority of schools/colleges at a minimum offer research experiences, little has been published regarding the impact of these experiences on the participating students or faculty.

In order to improve our students’ ability to be successful in securing their desired types of jobs or post-graduate training programs [21, 22], the Bill Gatton College of Pharmacy, a Doctoral/Research University classified institution, has placed an increased emphasis on student engagement in the research process at the administrative, faculty, and student levels. A previous article describes the development of one research elective course at the college in which students can choose to participate [23]. Students may choose to take a single research elective, or they may participate in a research concentration which requires them complete an application and oversight process, 12 credit hours of research courses, and denotes “Award for Excellence in Research” on their transcript.

At the time this survey was conducted, 16 faculty members in both the Departments of Pharmaceutical Sciences and Pharmacy Practice offer similar second (P2)- and third (P3)-professional year research elective courses, and/or a fourth-professional year (P4) elective Advanced Pharmacy Practice Experience (APPE) during which students can write a research manuscript based on research conducted during the P2 and/or P3 year(s). Some students are even paired with faculty who are residency directors that intentionally team the students with a post-graduate year 2 (PGY2) trainee for specific mentoring in clinical residency-type research.

Faculty members independently set forth requirements for successful completion of their research electives and thus student experiences vary based on faculty mentor, but in general for every credit hour, students should devote 2 h per week to their research project. A standardized syllabus is used for all research electives which includes a section completed by the faculty member, the student, and the department chair that outlines the expectations and requirements of each individual experience. Students seeking the “Award for Excellence in Research” designation must complete additional standardized requirements which are overseen by a college level committee comprised of faculty engaged in student research. Research allocation varies from faculty to faculty as the time allocation for research is set by the department chair in collaboration with the faculty and is dependent on factors such as level of funding. It is important to note that most faculty, particularly clinical/non-tenure track faculty in the Department of Pharmacy Practice, are not externally funded. The Department of Pharmacy Practice only has one tenure track faculty member who is supported by extramural funding. The college encourages participation in research, though, and the students’ engagement in research through the promotion and tenure process.

The purpose of this article is to describe the impact on students and faculty of participation in the research elective from its inception (2009) through the fall semester of the 2013–2014 academic year in our non-research intensive institution. To quantify outcomes of participation in a research elective sequence, college records from the office of Academic Affairs were retrospectively analyzed and all faculty members were surveyed via email to quantify outcomes specific to research elective(s) offered within the College, including poster/podium presentations given, peer-reviewed manuscripts published, and funding support obtained. Faculty are encouraged to only include students as authors who meet guidelines set forth by the International Committee of Medical Journal Editors. There were no incentives to the participants in the survey. All faculty members responded to the survey. The information related to this project was reviewed by the Institutional Review Board (IRB) Medical Campus at East Tennessee State University and the determination was that the project did not need IRB review and IRB approval.

## Results

Since fall of 2009 (the first semester during which a research elective was offered), 145 students (approximately 30 % of all P2 and P3 students) have taken at least one research elective course for credit. Our college has a class size of approximately 80 students per year. The percent of students enrolling in research elective courses has increased over time from 30 % of the Class of 2012 to 49 % of the Class of 2015. Fifty-four percent of the Class of 2016 has either completed or planned to complete a research elective course through the fall of 2014. All faculty who had taken research students responded to the survey (100 % response rate).

Sixty students enrolled in the research elective series for more than one semester. Of these, 11 students are on track to complete the 12-credit sequence by the time of their graduation next May in the Class of 2016. Additionally, some students conducted research without credit; therefore, the outcomes described herein are conservative. From 2009 to 2013, students presented 81 posters and gave 14 podium presentations at local or national conferences. In addition, faculty members involved in this course have published 15 articles in peer-reviewed journals in which students met criteria to be included as authors (Table 1). Fifty-three percent of posters and 14 % of podium presentations were disseminated on the national level. Also, a survey of research elective students conducted by one faculty member noted positive feedback from the students, including improved problem solving ability, knowledge, scientific writing ability, and application of theoretical knowledge gained in the classroom to research areas [23].

**Table 1:** Scholarly activities related to pharmacy students

Number of abstract(s) presented in collaboration with students				Number of article(s) published or accepted in peer-reviewed journals in collaboration with students	Number of funded projects on which pharmacy students worked	
Poster		Podium			Internal funding	Extramural funding
Local	National	Local	National			
38	43	12	2	15	19	8

Fourty percent of clinical (non-tenure) track faculty have coordinated one or more research elective offerings, and 82 % of tenured/tenure-track faculty have done the same, which indicates a strong interest in being involved in the research process in both Pharmaceutical Sciences and Pharmacy Practice departments. On average, three additional students per academic year, not including students who are already enrolled in and continue to pursue courses in the research elective sequence, take a research elective course. Whereas a majority of students participate in research activities supported by intramural funds (provided at the departmental level for new faculty or from the university level via intramural grants from the research development committee) or conducted in kind, 8 students have participated in externally funded research, 19 in internally funded projects, and the remainder conducted in kind.

## Conclusions

Limitations of this research include an unblinded survey instrument and the potential for recall bias since there has been a period of several years since the initial courses were offered in 2009. Given that all 16 participating faculty members responded to the survey, it is more likely that the results are accurate. Moreover, when looking at objective data, such as number of participants, these were taken from college records and not dependent on participant recall. Obviously though participants are invested in this training and there is the potential for desirability bias; however, since all faculty participated and the results were consistent, the researchers feel the results are credible. Additionally, we did not collect the individual number of students involved with each publication or poster so we cannot comment on the frequency of dissemination of research findings for those students involved.

Students benefit from the research process through increased engagement and critical thinking skills as well as better positioning for desired positions post-graduation. One potential drawback is that engaging students in scholarly activities requires sufficient faculty time, adequate resources, and appropriate faculty expertise [11]. Pearson and Albon indicate institutional support is a major factor in success of an educational research program [24]. Our culture supports and fosters scholarship through provision of research funds and reasonable research time allocation in addition to consideration of time invested in student research during the promotion and tenure process. As a relatively new college and one not considered research intensive, administration, faculty, and students could easily justify minimization of the research/scholarship leg of the College’s academic stool, although this would be inconsistent with the approach desired for colleges and schools of pharmacy by ACPE. Another challenge is to increase the number of clinical/non-tenure track faculty engaged in this process. These individuals have very limited amount of workload directed towards research. Administrators could facilitate this by increasing research and scholarship workload allotments for clinical/non-tenure track faculty and by continuing to reward student research mentorship in the promotion and tenure process. In fact, at our college, this will be introduced as a discussion point for a modification of the Department of Pharmacy Practice promotion and tenure document. While difficult to capture in a tangible manner, we perceive these experiences to be beneficial to current students, alumni, faculty, the College, and the profession as a whole.

Students engaged in the research process develop skill sets and critical thinking skills that will serve them and the profession well, irrespective of practice or research setting. Kehrer and Svennson recently stated, “If colleges and schools of pharmacy are to meet the goals of developing inquisitive and creative graduates who are lifelong learners and change agents, they must lead by example and provide scholarly opportunities to stimulate creativity in their students” [15].

In summary, this article provides a compelling argument that involving student pharmacists in the research process is feasible for pharmacy faculty, particularly when they have the support of college or school administration. We posit, and ACPE supports, that this is not only a responsibility of research intensive institutions, but of all schools/colleges. We therefore encourage all schools/colleges to consider provision of such opportunities and to assess and share associated outcomes in an effort to advance best practices in the engagement of pharmacy students in research and scholarship activities.
